# Cyclotron production of ^43^Sc and ^44g^Sc from enriched ^42^CaO, ^43^CaO, and ^44^CaO targets

**DOI:** 10.3389/fchem.2023.1167783

**Published:** 2023-04-26

**Authors:** Kaelyn V. Becker, Eduardo Aluicio-Sarduy, Tyler Bradshaw, Samuel A. Hurley, Aeli P. Olson, Kendall E. Barrett, Jeanine Batterton, Paul A. Ellison, Todd E. Barnhart, Ali Pirasteh, Jonathan W. Engle

**Affiliations:** ^1^ Department of Medical Physics, University of Wisconsin, Madison, WI, United States; ^2^ Department of Radiology, University of Wisconsin, Madison, WI, United States

**Keywords:** scandium, positron emission tomography (PET), cyclotron, radionuclide production, phantom imaging

## Abstract

**Introduction:** ^43^Sc and ^44g^Sc are both positron-emitting radioisotopes of scandium with suitable half-lives and favorable positron energies for clinical positron emission tomography (PET) imaging. Irradiation of isotopically enriched calcium targets has higher cross sections compared to titanium targets and higher radionuclidic purity and cross sections than natural calcium targets for reaction routes possible on small cyclotrons capable of accelerating protons and deuterons.

**Methods:** In this work, we investigate the following production routes via proton and deuteron bombardment on CaCO_3_ and CaO target materials: ^42^Ca(d,n)^43^Sc, ^43^Ca(p,n)^43^Sc, ^43^Ca(d,n)^44g^Sc, ^44^Ca(p,n)^44g^Sc, and ^44^Ca(p,2n)^43^Sc. Radiochemical isolation of the produced radioscandium was performed with extraction chromatography using branched DGA resin and apparent molar activity was measured with the chelator DOTA. The imaging performance of ^43^Sc and ^44g^Sc was compared with ^18^F, ^68^Ga, and ^64^Cu on two clinical PET/CT scanners.

**Discussion:** The results of this work demonstrate that proton and deuteron bombardment of isotopically enriched CaO targets produce high yield and high radionuclidic purity ^43^Sc and ^44g^Sc. Laboratory capabilities, circumstances, and budgets are likely to dictate which reaction route and radioisotope of scandium is chosen.

## 1 Introduction

An increasingly popular approach to cancer therapy is combining chemically similar pairs of radionuclides as radiolabels of diagnostic and therapeutic drugs (“theranostics”). Positron emission tomography (PET) of ^68^Ga- (t_1/2_ = 67.71 min; β^+^_Eavg_ = 829.5 keV; β^+^_intensity_ = 88.91%) labeled somatostatin analogs and prostate-specific membrane antigen targeting agents has been used to monitor response to ^177^Lu- (t_1/2_ = 6.644 days; β^−^_EAvg_ = 133.6 keV; β^-^_intensity_ = 100.0%) based treatment (Weineisen, et al., 2015; Grubmüller, et al., 2018; Heinzel, et al., 2019), to select potential patients for therapy (Gains, et al., 2011), and to detect lesions not found on ^177^Lu single photon emission tomography (SPECT) (Sainz-Esteban, et al., 2012). However, the high average positron energy and short half-life of ^68^Ga limit its clinical application. Because ^68^Ga’s half-life is too short for off-site transport, clinics require an on-site cyclotron or a^68^Ge/^68^Ga generator to provide patient doses.

Radioisotopes of scandium are promising alternatives to ^68^Ga. There are three clinically interesting radioisotopes of scandium: ^43^Sc (t_1/2_ = 3.891 h; β^+^_Eavg_ = 476 keV; β^+^_intensity_ = 88.1%), ^44g^Sc (t_1/2_ = 3.97 h; β^+^_Eavg_ = 632 keV; β^+^_intensity_ = 94.27%), and ^47^Sc (t_1/2_ = 3.349 days; β^−^_Eavg_ = 162 keV; β^-^_intensity_ = 100.0%) (National Nuclear Data Center, Brookhaven National, 2022). Both ^43^Sc and ^44g^Sc emit positrons with lower mean energies than ^68^Ga, have high positron branching ratios, and are chemically identical to the therapeutic radionuclide ^47^Sc. Additionally, the ionic radius and coordination behavior of scandium are better matched to lutetium than gallium (Robert, 1976); its positron-emitting radioisotopes are viable diagnostic analogues of ^177^Lu in radiopharmaceuticals. Proton bombardment of natural metallic calcium targets produces ^44g^Sc (Severin, et al., 2012), but long-lived (t_1/2_ = 44 h–84 days) radionuclidic impurities (^46^Sc, ^47^Sc, and ^48^Sc) are co-produced due to the many stable isotopes of calcium (^40^Ca, 96.941%; ^42^Ca, 0.647%; ^43^Ca, 0.135%; ^44^Ca, 2.086%; ^46^Ca, 0.004%; ^48^Ca, and 0.187%). Producing ^43^Sc and ^44g^Sc from isotopically enriched calcium minimizes the presence of long-lived radioisotopes of scandium. This work investigates the following reactions on isotopically enriched calcium targets: ^42^Ca(d,n)^43^Sc, ^43^Ca(p,n)^43^Sc, ^43^Ca(d,n)^44g^Sc, ^44^Ca(p,n)^44g^Sc, ^44^Ca(p,2n)^43^Sc, and ^44^Ca(d,2n)^44g^Sc.

There are several methods for separating ^43/44g^Sc from calcium targets. Following target dissolution, extraction chromatography resins such as UTEVA^®^ (Valdovinos, et al., 2015), N,N,N′,N′-tetra-n-octyldiglycolamide (DGA) (Alliot, et al., 2015), and hydroxamate (Severin, et al., 2012) isolate ^43/44g^Sc from the calcium target material. Additionally, adding ion exchange resins such as Dowex^®^ 50WX8 in combination with extraction chromatography resin such as DGA further removes trace metal impurities (Rane et al., 1966; Müller, et al., 2013; van der Meulen, et al., 2015).

Irradiating titanium targets with protons also produces ^43/44g^Sc (Loveless, et al., 2021; Şekerci̇ et al., 2023). Like the calcium targets, ^nat^Ti targets are feasible for producing ^43/44g^Sc, but production of high radionuclidic purity ^43/44g^Sc requires enriched titanium (^46^Ti and ^47^Ti) targets due to reactions on other stable isotopes of titanium (^46^Ti, 8.25%; ^47^Ti, 7.44%; ^48^Ti, 73.72%; ^49^Ti, 5.41%; ^50^Ti, 5.18%). The ^46^Ti(p,α)^43^Sc and ^47^Ti(p,α)^44g^Sc reaction routes have lower cross sections than proton-induced ^43/44g^Sc reactions on calcium target material, but ^46^Ti and ^47^Ti are cheaper than the enriched calcium material, and may also handle higher currents (Chernysheva, et al., 2021). Domnanich et al., 2017a, reported a 90–120 μAh, 15.1 ± 1.9 MeV proton irradiation of 10 mg of 97.0% ± 0.02% isotopically enriched ^46^TiO_2_ targets produced 1.17–1.22 MBq/μAh ^43^Sc with 98.2% ± 0.3% radionuclidic purity. Similarly, a 183 µAh, 12.0 ± 2.3 MeV proton irradiation of 10 mg of 57.9% ± 1.8% isotopically enriched ^43^CaCO_3_ (12.36% ^44^CaCO_3_) produced 2.62 MBq/μAh ^43^Sc with 66.2% ± 1.5% radionuclidic purity (33.3% ± 1.5% ^44g^Sc) (Domnanich, et al., 2017b). Production of the 33.3% ± 1.5% ^44g^Sc was the result of the (p,n) reaction on the 12.36% ^44^CaCO_3_ present in the target material. The two routes achieved similar radionuclidic purities with the ^46^TiO_2_(p,α)^43^Sc reaction producing 99.7% ± 0.5% ^43/44g^Sc and the ^43^CaCO_3_(p,n)^43^Sc reaction producing 99% ± 2% ^43/44g^Sc at end of bombardment.

For sites without accelerators, a generator of ^44g^Sc’s long-lived parent ^44^Ti (t_1/2_ = 60.0 year) (National Nuclear Data Center, Brookhaven National, 2022) has been developed (Gajecki and Marino., 2023; under review; Schumann et al., 2007; Loktionova, et al., 2010; Filosofov, et al., 2010; Pruszyński and Loktionova, 2010; Radchenko, et al., 2016; Radchenko, et al., 2017; Rane, et al., 2015; Kerdjoudj, et al., 2016; Müller, et al., 2018; Roesch, 2012)^1^. ^44^Ti’s long half-life, the potential for high efficiency elutions (>97%) (Filosofov, et al., 2010), and the lack of ^44m^Sc make this system appealing. For a^44^Ti/^44g^Sc generator to be clinically viable, the generator must have low ^44^Ti breakthrough, ^44g^Sc elutions suitable for labeling (low volume, high activity concentration), and consistent elution results. Several published generator designs use AG 1-X8 anion exchange resins which result in large elution volumes and ^44^Ti breakthrough requiring reverse elution modes (Filosofov, et al., 2010; Pruszyński and Loktionova, 2010; Roesch, 2012; Kerdjoudj, et al., 2016). More recent designs use ZR hydroxamate resin which may increase generator lifetimes due to the increased number of elutions achieved before ^44^Ti breakthrough is observed (Gajecki and Marino, 2023). A ^47^Ca/^47^Sc generator system for the therapeutic radioisotope of scandium is also in development (Mausner, et al., 1998; Rane, et al., 2015; Starovoitova, et al., 2015; Misiak, et al., 2017; Carzaniga et al., 2019; Pawlak, et al., 2019). However, ^47^Ca production requires enriched ^48^Ca which is expensive and not widely available.

It is also possible to produce ^47^Sc through proton bombardment of titanium targets [^nat^Ti(p,x)^43^,^44g^,^47^Sc, ^48^Ti(p,2n)^47^Sc, ^50^Ti(p,α)^47^Sc]. These reactions suffer from co-production of ^46^Sc and ^48^Sc at high energies (>30–35 MeV) so lower proton energies are ideal (Chernysheva, et al., 2021). There are also two reaction routes to ^47^Sc from calcium target material: ^48^Ca(p,2n)^47^Sc and ^44^Ca(α,p)^47^Sc (Minegishi, et al., 2016; Sitarz, et al., 2018; Carzaniga et al., 2019; Wibowo et al., 2021; Aikawa, et al., 2022). However, neither ^48^Ca target material nor alpha accelerators are widely accessible, and these reactions routes will likely not have widespread use. Other alternative production paths to produce ^47^Sc include proton bombardment of natural vanadium targets [^nat^V(p,x)^47^Sc] (Jafari, et al., 2019; Barbaro, et al., 2021; De Nardo, et al., 2021), photonuclear reactions via electron linear accelerators [^48^Ti(γ,p)^47^Sc and ^51^V(γ,α)^47^Sc] (Kazakov, et al., 2012; Rotsch, et al., 2018; Loveless, et al., 2019; Aliev, et al., 2020; Snow, et al., 2021) and reactor production with fast [^46^Ca(n,γ)^47^Ca → ^47^Sc] or thermal [^47^Ti(n,p)^47^Sc] neutrons (Domnanich, et al., 2017b; Jalilian, et al., 2021).

Once radiochemically separated from the target material, ^44g^Sc has been successfully complexed and exhibited high *in-vivo* stability with the commonly used chelator 1,4,7,10-tetraazacyclododecane-1,4,7,10-tetraacetic acid (DOTA) (Huclier-Markai, et al., 2014). As an alternative to DOTA, novel chelators designed and optimized for scandium include the heptadendate AAZTA ligands, the nonmacrocyclic H_4_pypa chelator, and the triaza-macrocycle-based picolinate-functionalized chelator H_3_mpatcn that can be labeled with scandium at room temperature (Li, et al., 2020a; Vaughn, et al., 2020a; Li, et al., 2020b; Vaughn, et al., 2020b; Fersing, et al., 2022). *In vivo* and *in vitro* studies have investigated ^44g^Sc labeled with DOTA-based peptides (Hernandez, et al., 2014; van der Meulen, et al., 2015; Eppard, et al., 2017; Singh, et al., 2017) and conjugated AAZTA-PSMA peptides (Sinnes, et al., 2020; Ghiani, et al., 2021). Additionally, ^44g^Sc-labeled antibody fragments detected 4T1 murine breast cancer tumors and U87MG human glioblastoma tumors in murine models (Chakravarty, et al., 2014; Li, et al., 2020b).

Preclinical and clinical PET/CT studies have compared the image quality of ^44g^Sc (Bunka, et al., 2016; Ferguson, et al., 2019; Rosar, et al., 2020; Lima, et al., 2021) and, less frequently, ^43^Sc (Lima, et al., 2021), to conventional PET radionuclides. These studies have shown that both ^43^Sc and ^44g^Sc have favorable contrast and image resolution compared to ^68^Ga. ^43^Sc’s, and ^44g^Sc’s relatively longer half-lives enable transportation to nearby clinics, increasing their accessibility.

Currently, enriched calcium is only available as CaCO_3_, a suboptimal material for cyclotron irradiation due to CaCO_3_’s low thermal conductivity and decarboxylation (and subsequent release of CO_2_ at high temperatures) in beam. Additionally, while previous works have investigated cross section measurements of various proton- and deuteron-induced reactions on calcium, there is a lack of comprehensive studies comparing the various radionuclidic purities and production yields for small cyclotron-produced ^43^Sc and ^44g^Sc. The goal of this work is to investigate and optimize the possible production routes of both ^43^Sc and ^44g^Sc using enriched calcium targets irradiated on a small cyclotron capable of accelerating 16 MeV protons and 8 MeV deuterons and compare their contrast on clinical PET/CT scanners.

## 2 Materials and methods

### 2.1 Chemicals

Enriched ^42^CaCO_3_, ^43^CaCO_3_, and ^44^CaCO_3_ materials were obtained from the Department of Energy National Isotope Development Center with isotopic purities of 93.58%, 83.93%, and 98.89%, respectively (Table 1). Solutions were prepared with optima grade HCl and HNO_3_ purchased from Fisher Chemical and 18 MΩ·cm^−1^ water. N,N,N′,N′-tetrakis-2-ethylhexyldiglycolamide (branched DGA) resin was purchased from Eichrom. DOTA chelator and DOTA-(Tyr^3^)-octreotate (DOTA-TATE) were purchased from Macrocyclics.

**TABLE 1:** Isotopic composition of enriched CaCO_3_ material received from the DOE National Isotope Development Center.

Calcium isotope	^42^CaCO_3_	^43^CaCO_3_	^44^CaCO_3_
^40^Ca	5.24 ± 0.02	10.13 ± 0.08	1.04 ± 0.01
^42^Ca	93.58 ± 0.05	0.78 ± 0.006	0.036 ± 0.003
^43^Ca	0.33 ± 0.02	83.93 ± 0.10	0.026 ± 0.002
^44^Ca	0.84 ± 0.01	5.06 ± 0.03	98.89 ± 0.02
^46^Ca	<0.01	<0.001	<0.001
^48^Ca	0.01 ± 0.01	0.09 ± 0.001	<0.001

### 2.2 Target fabrication and cyclotron irradiation

**FIGURE 1:**
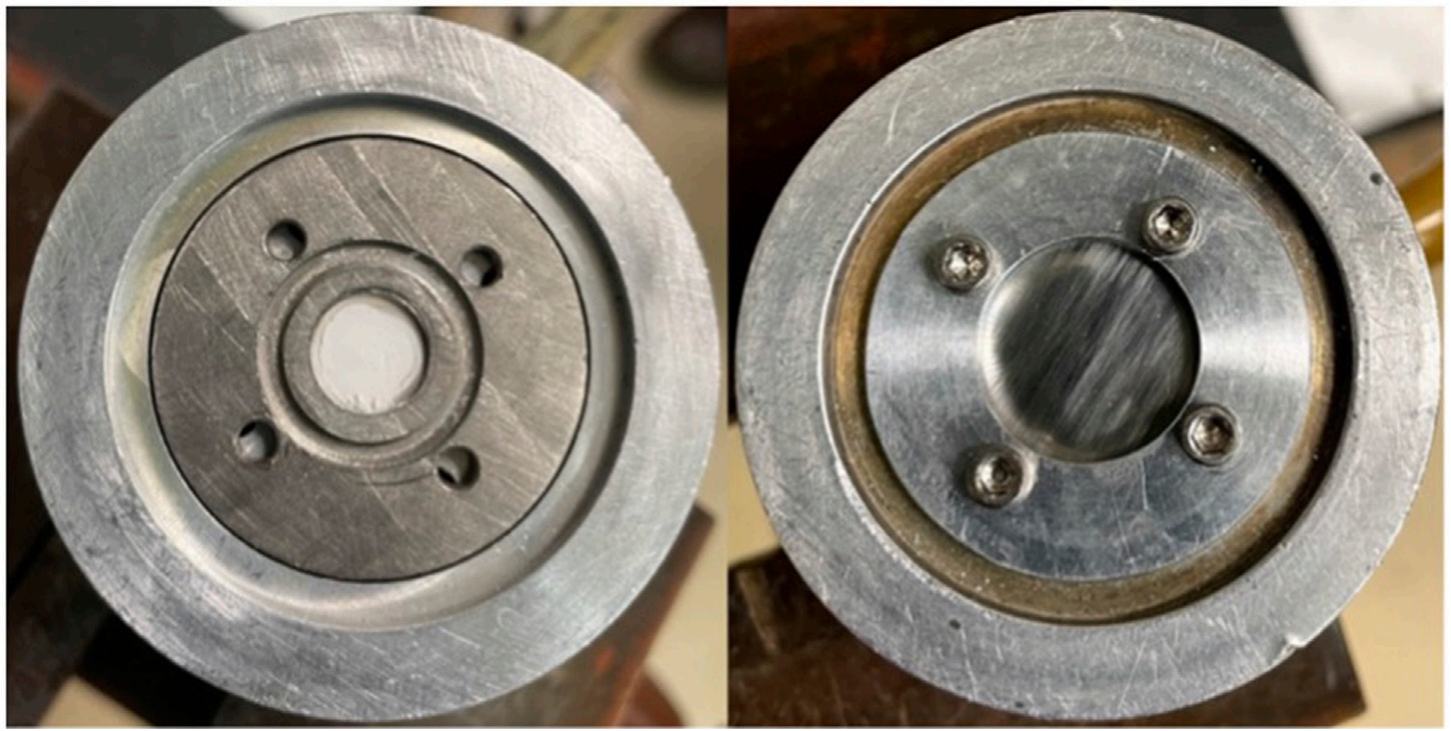
Niobium crucible containing ∼60 mg pressed CaO powder (left) and covered with 50 μm niobium cover foil (right).

**FIGURE 2:**
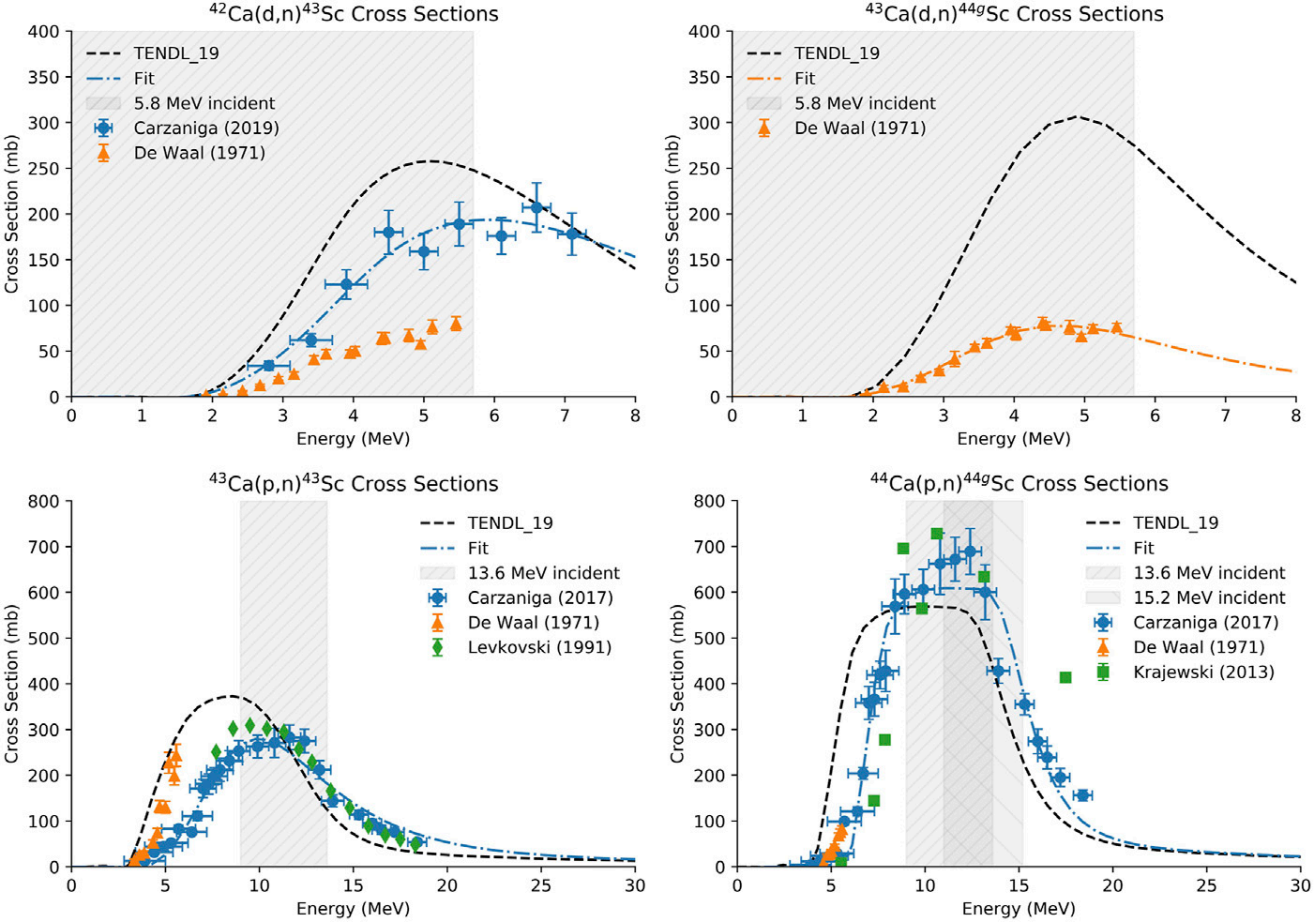
Cross sections for the ^42^Ca(d,n)^43^Sc, ^43^Ca(d,n)^44g^Sc (top) (Carzaniga, et al., 2019; de Waal, et al., 1971a; Koning et al., 2012; de Waal, et al., 1971), ^43^Ca(p,n)^43^Sc, and ^44^Ca(p,n)^44g^Sc (bottom) reactions. The energy range investigated is highlighted in gray and a fit of the experimental data is shown in blue.

Enriched CaCO_3_ targets have been reported to withstand proton currents up to 50 μA when kept relatively thin (0.1–0.2 mm) and of low mass (<10 mg) (van der Meulen, et al., 2015). However, this material is very hygroscopic and readily absorbs water from the atmosphere. Additionally, when sufficiently heated in beam, CaCO_3_ decarboxylates, forming CaO, offgassing CO_2_, potentially damaging the target, and reducing accelerator beam transmission. A transition from preclinical to clinical scale productions suggests need for larger target masses and/or higher beam currents to increase yield. To compensate for this increased power deposition, we used CaO as an alternative target material. CaCO_3_ was decarboxylated to CaO in a vertical tube furnace (Thermansys) heated in an inert atmosphere to 900°C–950°C. A graphite crucible containing approximately 150–300 mg of CaCO_3_ was lowered into a flat-bottomed quartz tube (the graphite crucible facilitated removal of the powder) and a compression fitting with an argon gas line was connected to the quartz tube to prevent degradation of the graphite crucible during heating. The crucible and quartz tube were placed in the preheated furnace under argon gas flow for 60–90 min. To confirm complete thermal decomposition of the CaCO_3_ material, the mass of the crucible and powder was measured before and after heating, and the decomposition of the graphite crucible was accounted for. Following the decarboxylation procedure, the CaO was quickly pressed into a 9.5 mm diameter niobium crucible with a hydraulic press (250 kg/cm^2^) and covered with a thin niobium cover foil to confine the target material and degrade the incident proton/deuteron energy (Figure 1). For the ^44^Ca(p,n)^44g^Sc reaction, two incident energies were used. A 50 µm thick foil degraded the incident 16 MeV protons to 15.2 MeV and a 150 μm thick niobium cover foil degraded the incident energy to 13.6 MeV (calculated using SRIM) (Ziegler and Ziegler, 2010). Additionally, the 50 µm thick foil degraded the incident 8 MeV deuterons to 5.8 MeV. These energies were chosen to minimally degrade the 16 MeV proton beam (incident energy of 15.2 MeV) and 8 MeV deuteron beam (incident energy of 5.8 MeV) and to cover the peak of the cross-section curve (incident energy of 13.6 MeV). Figure 2 shows experimentally measured cross sections along with the energy range investigated. The target was then loaded on the University of Wisconsin, Madison’s GE PETtrace cyclotron or placed under vacuum in a furnace preheated to >100°C to keep the target from absorbing moisture. For the CaCO_3_ targets, 75–150 mg CaCO_3_ was first heated in a polypropylene centrifuge tube at 90°C for 30 min to ensure that the material was dry and then pressed directly into the niobium crucible. All targets were irradiated for 15°min to 1 h with 5–20 μA protons or 3–10 μA deuterons with water jet cooling on the back of the target.

To compare current tolerances, pressed powder ^nat^CaCO_3_ and ^nat^CaO targets of equal thickness were irradiated with 13.6 MeV protons. Cyclotron vacuum pressure was monitored during irradiation, and peak and stabilized pressures were recorded as the current was increased in 1 μA steps.

### 2.3 HPGe spectrometry

Targets were assayed via High Purity Germanium (HPGe) gamma spectrometry with an aluminum-windowed detector (Ortec) coupled to a Canberra Model C1519 research amplifier and multi-channel analyzer (full-width half-maximum at 1,333 keV = 1.8 keV) to quantify radionuclide activities 10–30 min after irradiation. ^241^Am, ^133^Ba, ^152^Eu, ^137^Cs, and ^60^Co check sources traceable to NIST were used to energy and efficiency calibrate the detector. Full targets were measured 200–400 cm from the detector face to quantify ^43/44g/44m^Sc activity, and longer-lived radioisotopes of scandium (^46^Sc, ^47^Sc, and ^48^Sc) were quantified through 250 μL aliquots of the dissolved target (5 mL) measured overnight approximately 20 cm from the detector face. To determine end of bombardment (EoB) yields of long-lived scandium radioisotopes, the activity of ^43^Sc or ^44g^Sc quantified in the aliquot provided a correction factor to obtain ^46^^,^^47^^,^^48^Sc full target activity.

### 2.4 Radiochemical isolation of ^43/44g^Sc

**FIGURE 3:**
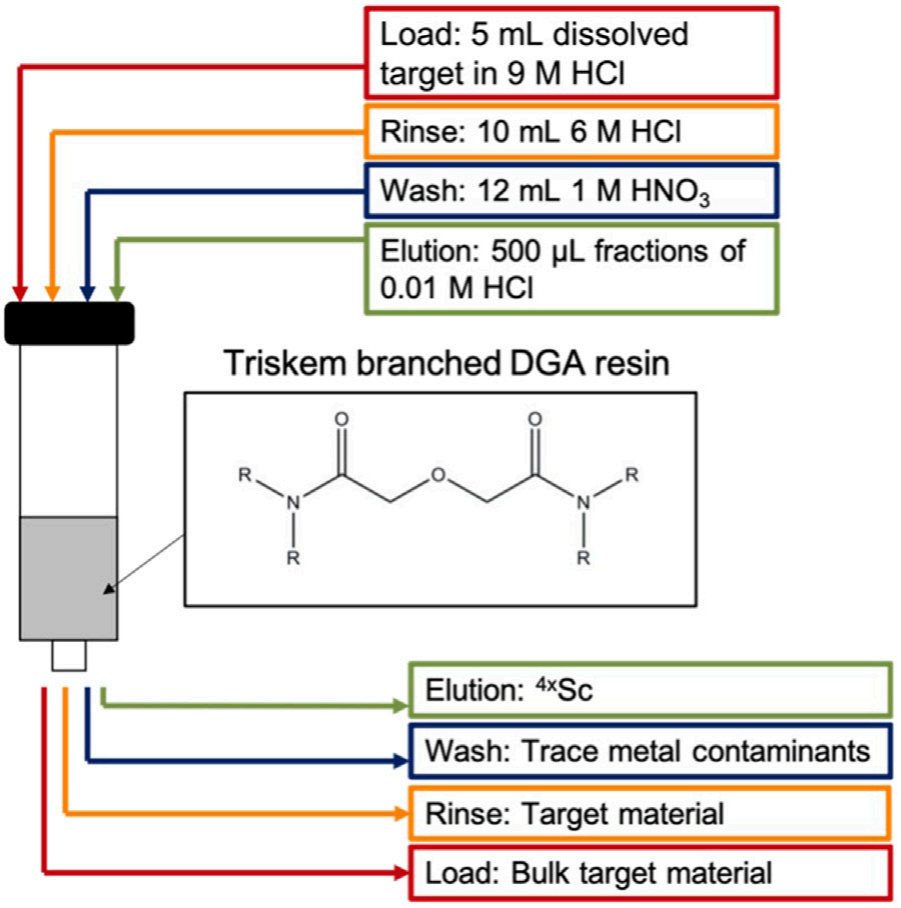
Ca/Sc separation strategy utilizing branched DGA resin.

Targets were dissolved in 5 mL 9 M HCl heated at 80°C with magnetic stir bar agitation for 30 min. Radiochemical separation of ^43/44g^Sc from ^42/43/44^CaO target material used single-column extraction chromatography with branched DGA (132 ± 67 mg, 5.5 mm column diameter) resin pre-conditioned with 3 mL 0.01 M HCl followed by 5 mL 9 M HCl (Figure 3). The 5 mL dissolved target solution was loaded on to the conditioned resin using a peristaltic pump (1.6 mL/min) and collected for target recycling along with a 10 mL rinse of 6 M HCl. To remove trace metals and reduce the acidity in the column, a 12 mL 1 M HNO_3_ rinse was performed and ^43/44g^Sc was subsequently eluted with four 500 μL fractions of 0.01 M HCl (Alliot, et al., 2015; Aluicio-Sarduy, et al., 2018). Activities were assayed in a Capintec CRC-15R dose calibrator (^43^Sc setting #825; ^44g^Sc setting #938). Capintec dose calibrator settings were taken from the user’s manual and confirmed via cross-calibration with HPGe spectroscopy.

### 2.5 Recovery of enriched calcium

The enriched calcium target material was recovered through precipitation from the 5 mL load and 10 mL rinse solutions. The calcium was precipitated as CaCO_3_ through careful drop-wise addition of saturated (NH_4_)_2_CO_3_ solution (approximately 30 mL), allowing 10–30 s for the solution to settle and CO_2_ to be released between drops of (NH_4_)_2_CO_3_. The precipitate was then centrifuged, decanted, and dried under argon at 120°C. The dried CaCO_3_ could then be decarboxylated to CaO for future irradiations following the same procedure described above.

### 2.6 Apparent molar activity measurements and trace metals analysis

Trace metals analysis on the eluted ^43/44g^Sc solutions was performed by microwave plasma-atomic emission spectrometry (MP-AES) using an Agilent Technologies 4,200 spectrometer. Calibration standards containing 10 ppb to 100 ppm of Fe, Zn, Cu, and Ca were prepared in 0.01 M HCl solutions from 1,000 mg/L standards purchased from Sigma-Aldrich.

The apparent molar activity of the eluted ^43/44g^Sc solutions was determined through titration by reaction with varying concentrations of DOTA chelator. Polypropylene centrifuge tubes containing increasing concentrations of DOTA dissolved in 18 MΩ water (100 μL, 0–100 μg/mL) were prepared and small aliquots (10–15 μL, 3.7 ± 0.04 MBq) of ^43/44g^Sc eluate were added to each vial. Additionally, 2–5 μL of 1.5 M NaOAc (pH = 4.5) buffer were added to each vial to reach a final pH between 4–4.5. The solutions were then vortexed and heated on a shaker plate at 80°C and 300°RPM for 30 min. The percent ^43/44g^Sc complexed with DOTA was determined through thin layer chromatography (TLC) using aluminum backed silica gel plates (TLC Silica gel 60 F_254_, Sigma-Aldrich) in 50 mM ethylenediaminetetraacetic acid (EDTA). Plates were imaged with storage phosphor autoradiography (Packard Cyclone). Under these conditions, the radiolabeled ^43/44g^Sc-DOTA stayed at the origin (retention factor, *R*_*f*_ = 0) while the free ^43/44g^Sc travelled with the mobile front (*R*_*f*_ = 1)*.* The apparent molar activity (MBq/µmol) of ^43/44g^Sc with DOTA was found by dividing the activity of ^43/44g^Sc in each vial by twice the amount of DOTA required to complex 50% of the activity.

Radiolabeling experiments were also performed with the DOTA-based peptide DOTA-TATE following the same radiolabeling conditions listed above for DOTA. Briefly, 100 μL ^43/44g^Sc (37 ± 5 MBq) was vortexed with 2–3 μL DOTA-TATE (4.3–5.7°nmol, 0.2 mCi/nmol; 7.14°MBq/nmol) and 2–5 μL 1.5 M NaOAc pH 4.5 buffer and heated to 80°C while shaking at 300°RPM for 15–30 min. Percent complexation was determined through radio-TLC and high-performance liquid chromatography (HPLC) using a C18 column (4.6 × 250 mm, Dionex 5 μm 120 Å; ThermoFisher) and a water (0.1% trifluoroacetic acid, TFA) to acetonitrile gradient (5% MeCN, 0–3.5°min; 5%–35% MeCN, 3.5–13°min; 35%–90% MeCN, 13–15°min; 90% MeCN, 15–19°min; 90%–5% MeCN, 19–20°min; 5% MeCN, 20–25 min) flowing at 1 mL/min.

### 2.7 Clinical phantom scans

In this study, ^43^Sc, ^44g^Sc, and a mixture of ^43^Sc and ^44g^Sc (^43/44g^Sc) were compared to other positron-emitting radionuclides (^18^F, ^68^Ga, and ^64^Cu) in a clinical Derenzo phantom (acrylic) purchased from Phantech (Madison, WI). The University of Wisconsin, Madison’s GE PETtrace cyclotron was used to produce ^43^Sc [^42^CaO(d,n)^43^Sc], ^44g^Sc [^44^CaO(p,n)^44g^Sc], ^18^F [H_2_^18^O(p,n)^18^F], and ^64^Cu [^64^Ni(p,n)^64^Cu]. The mixture of ^43/44g^Sc (88% ^43^Sc, 12% ^44g^Sc by activity at end of chemistry) was obtained through 13.6 MeV proton bombardment of 83.93% isotopically enriched ^43^CaO targets containing 5.06% ^44^Ca. ^68^Ga was eluted from a GalliaPharm ^68^Ge/^68^Ga generator (5 mL, 0.1°M HCl, Eckert and Ziegler, Inc.) at the University of Wisconsin, Madison radiopharmaceutical production facility. Table 2 lists the physical decay characteristics of the radionuclides used in this study and the effective half-life, positron intensity, and mean positron energy for the ^43/44g^Sc mixture.

**TABLE 2:** Physical decay characteristics and effective half-life and positron intensity of the radionuclides used in this study (National Nuclear Data Center, Brookhaven National, 2022).

	^43^Sc	^44g^Sc	^43/44g^Sc	^68^Ga	^64^Cu	^18^F
Half-life (h)	3.891	3.97	3.90	1.13	12.7	1.83
β^+^_intensity_	0.881	0.9427	0.888	0.8891	0.1749	0.9673
β^+^_Εavg_ (keV)	476	632	494.7	829.5	278	249.8
E_γ_ (keV) (I_γ_, %)	372.9 (22.5%)	1157.0 (99.9%)	--	1077.3 (3.22%)		

The Derenzo phantom consisted of 6 sections of rods with diameters of 3.5, 4.0, 4.5, 5.0, 5.5, and 6.0°mm, and was filled with activities shown in Table 3. Filled activities were increased for low positron intensity radionuclides (^64^Cu) and short-lived radionuclides (^68^Ga and ^18^F) to keep scan times consistent (under 15 min). The concentrated activity solutions were prepared by first mixing 250 mL 18 MΩ water, 200 μL Tween^®^ 80 (Sigma-Aldrich), 25 μL 6 M HCl (final pH 4.5), and then adding the radioactivity (2.74–19.97 MBq; 0.075–2.16 mCi). Tween^®^ was used to prevent bubbles from forming in the linear Derenzo phantom and the acid was added to prevent the radiometals from sticking to the walls of the phantom. Additionally, 2–3 drops of food coloring were added to facilitate detection of air bubbles when filling the small rods of the Derenzo phantom. Radioactivity was drawn into 1 mL syringes and measured in a Capintec CRC-15R dose calibrator before and after adding to the prepared solutions.

**TABLE 3:** Filled radioactivity derived from dose calibrator and HPGe recorded radioactivities and measured volumes.

Radionuclide	Filled activity concentration (kBq/mL)	Dose calibrator setting #
^43^Sc	13.04	825
^44g^Sc	10.97	938
^43/44g^Sc	15.43	825
^68^Ga	26.23	416
^64^Cu	79.87	055
^18^F	43.85	455

The filled phantom was scanned on a clinical GE Discovery 710 and GE Discovery MI PET/CT (GE Healthcare, Waukesha, WI) scanner. The GE Discovery 710 has 13,824 lutetium-yttrium oxyorthosilicate (LYSO) scintillator crystals (4.2 × 6.3 × 25 mm^3^) optically connected to photomultiplier tubes without a light-guide (Yoon et al., 2015). The axial field of view is 15.7 cm, and the transaxial field of view is 70 cm. The energy window is 425–650 keV with a coincidence window of 4.9 ns. The GE Discovery MI has 19584 LYSO scintillator crystals (3.95 × 5.3 × 25 mm^3^) connected to arrays of silicon photomultiplier (SiPM) detectors through light-guides (Hsu et al., 2017). The axial field of view is 20 cm, and the transaxial field of view is 70 cm. The energy and coincidence windows are identical to the GE Discovery 710 at 425–650 keV and 4.9 ns, respectively. Ten million coincidences were collected regardless of which radionuclide the phantom was filled with. Data were reconstructed using an OSEM-TOF reconstruction algorithm (VPFX; without point spread function) with a 256 × 256 matrix size and 16 subsets, 8 iterations on the GE Discovery MI and 24 subsets, 5 iterations on the GE Discovery 710.

Contrast was compared across the six radionuclides. To determine contrast, a transverse slice in the center of the phantom was selected, and a line profile was drawn from the center of one outermost rod to the center of the opposing outermost rod. The maximum and minimum values were then used to calculate the contrast:

$$C_{rod}=\frac{R_{\max}-R_{\min}}{R_{\max}+R_{\min}}$$

where $R_{\max}$ is the maximum value and $R_{\min}$ is the minimum value from the line profile.

## 3 Results

### 3.1 Target fabrication and cyclotron irradiation

**FIGURE 4:**
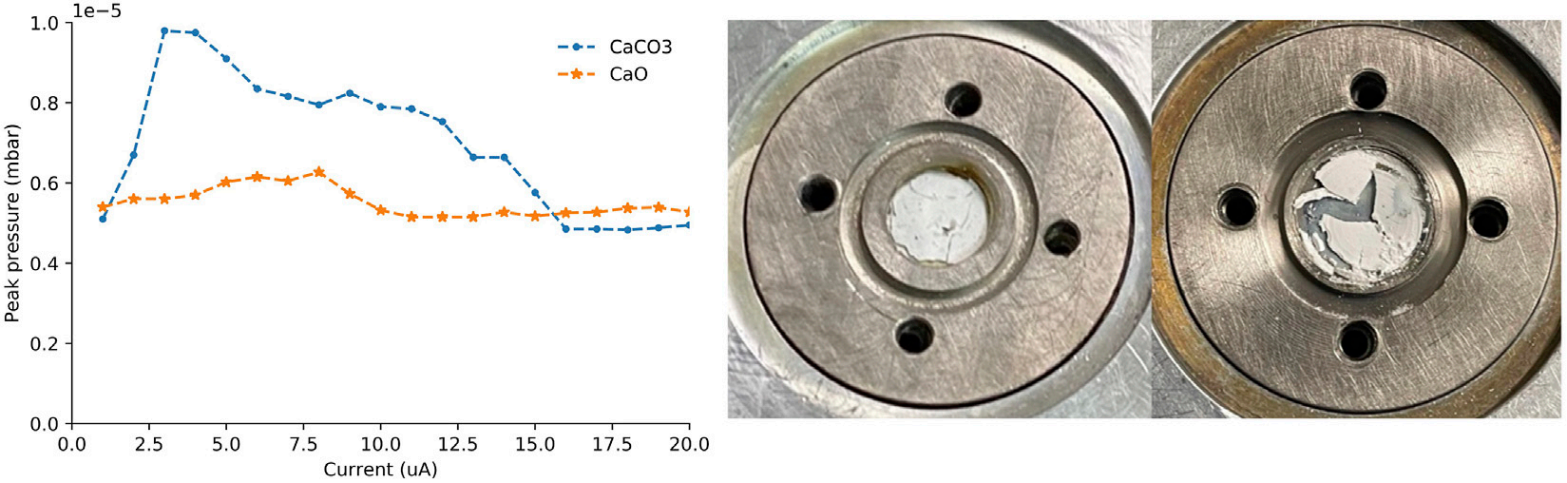
Peak pressure on the target as the current was increased (left). Pictures of the ^nat^CaO (middle) and ^nat^CaCO_3_ (right) targets immediately following 0–20 µA proton irradiation.

Pressed powder ^nat^CaCO_3_ and ^nat^CaO targets of approximately equal thickness (0.63 ± 0.02 mm; 158 ± 11 mg) were fabricated and irradiated with 13.6 MeV protons. The time required to reach 20 μA was 61 min for the ^nat^CaCO_3_ target and 23 min for the ^nat^CaO target. The ^nat^CaCO_3_ targets generated more gas load on the cyclotron’s vacuum system, slowing their progression to 20 μA. Following irradiation, the ^nat^CaO target sustained far less damage and did not suffer material losses while the ^nat^CaCO_3_ was fractured during irradiation (Figure 4).

For deuteron production yield measurements (*n* = 3 per route), ^42/43^CaO targets that were thick to the 5.8 MeV deuteron beam were fabricated (0.25 ± 0.01 mm; 61 ± 3 mg) (Figure 2). For proton production yield measurements (*n* = 3 per route), ^43/44^CaO targets that were 4.7 MeV thick to the 13.6 MeV beam (0.62 ± 0.02 mm; 149 ± 2 mg) were fabricated to capture the flat region of the cross section curve for the ^43^Ca(p,n)^43^Sc and ^44^Ca(p,n)^44g^Sc reactions (Figure 2) (Carzaniga, et al., 2017). Energy loss through the CaO targets was calculated using the Bethe-Bloch formula.

The ^44^Ca(p,2n)^43^Sc reaction has an energy threshold close to the maximum 16 MeV protons that the GE PETtrace can accelerate, so the thinner 50 μm niobium foil was used to minimally degrade the proton energy to 15.2 MeV (targets were kept the same mass and were consequently only 4.3 MeV thick for this reaction).

Targets were irradiated with either 5 μA protons or 3 μA deuterons with a total charge between 0.5 and 1.6 µAh. Production yields for the ^42^Ca(d,n)^43^Sc, ^43^Ca(p,n)^43^Sc, ^43^Ca(d,n)^44g^Sc, ^44^Ca(p,n)^44g^Sc, and ^44^Ca(p,2n)^43^Sc reactions are shown in Table 4. No ^43^Sc from the ^44^Ca(p,2n)^43^Sc was observed, indicating that 15.2 MeV is too close to the energy threshold for the reaction to produce significant quantities of ^43^Sc. This result agrees with previously reported data at 15.3 MeV (Carzaniga, et al., 2017).

**TABLE 4:** Observed production yields for the 5 routes investigated along with target masses and energy thickness (Carzaniga, et al., 2017; Carzaniga, et al., 2019)^*^ (de Waal, et al., 1971)^+^.

Reaction	Isotopic enrichment	Incident energy (MeV)	Target mass (mg)	Target thickness (MeV)	Production yield (mCi/μAh)	Yield corrected for beam intercept (mCi/μAh)	Predicted yield (mCi/μAh)	Radionuclidic purity (%)
^42^Ca(d,n)^43^Sc	93.58% ± 0.05%	5.8	62 ± 4	5.8	0.82 ± 0.05	1.49 ± 0.09	1.66*	99.36 ± 0.05
^43^Ca(p,n)^43^Sc	83.93% ± 0.10%	13.6	148 ± 1	4.6	6.19 ± 0.81	12.64 ± 0.92	15.41^*^	87.81 ± 0.03
^43^Ca(d,n) ^44g^Sc	83.93% ± 0.10%	5.8	61 ± 2	5.8	0.93 ± 0.08	1.69 ± 0.14	0.74^+^	98.08 ± 0.08
^44^Ca(p,n)^44g^Sc	98.89% ± 0.02%	13.6	150 ± 2	4.7	11.71 ± 1.09	23.92 ± 0.50	36.83^*^	99.71 ± 0.05
^44^Ca(p,2n)^43^Sc	98.89% ± 0.02%	15.2	151 ± 2	4.3	0	0	0	--
^44^Ca(p,n)^44g^Sc	98.89% ± 0.02%	15.2	151 ± 2	4.3	10.92 ± 1.34	22.60 ± 0.57	34.30^*^	99.51 ± 0.06

**FIGURE 5:**
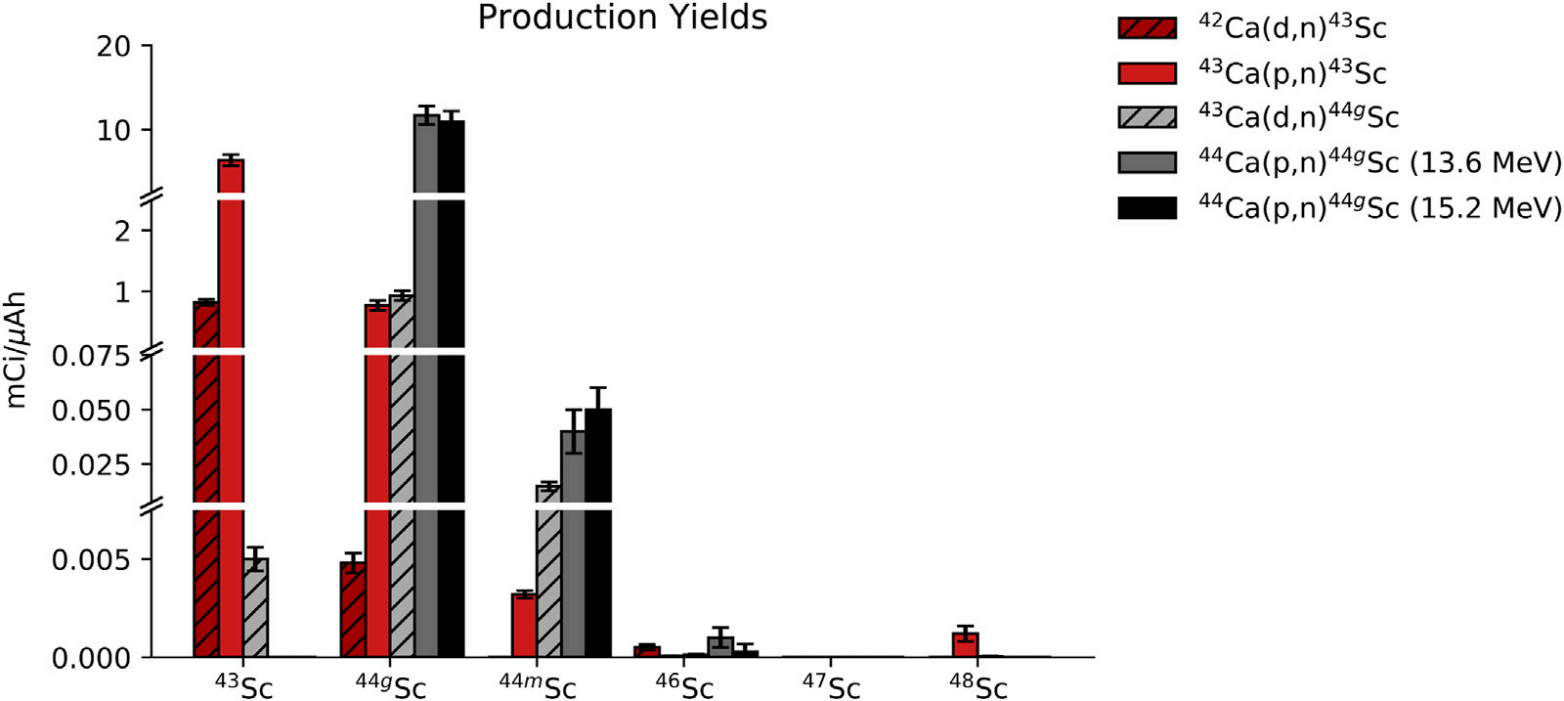
Activity of each scandium radioisotope that was present in the full target at EoB for each of the 5 reaction routes that were investigated.

Targets were also evaluated for radionuclidic purity via HPGe spectrometry. Figure 5 shows HPGe-measured yield decay-corrected to EoB (mCi/μAh) for each radioisotope of scandium, and the radionuclidic purity as a percentage of total activity decay-corrected to EoB is listed in Table 4 (*n* = 3 per reaction route).

The GE PETtrace beam spot on target is larger than the 9.5 mm target diameter, so the control system’s measured integrated charge on target (µA·h) overestimates integrated fluence on the ^42/43/44^Ca targets. To account for this, the niobium cover foil was used to determine the percentage of the beam that intercepted the target. After irradiation, a beam profile was obtained through autoradiography, and an ROI with a width of 9.5 mm was drawn and centered on the beam profile. A larger >20 mm diameter ROI was also drawn encompassing the entire beam spot. Additionally, four other 9.5 mm ROIs with an offset of up to 1.5 mm from center were drawn around the central region to determine the variance in beam intersection that may be caused from the beam drifting from the center. These values were independently measured for the proton and deuteron beams and were also evaluated before and after a cyclotron ion source puller electrode replacement that occurred during the measurement of these production yields. The measured beam intersections and the max difference between the offset regions are listed in Table 5 with a correction to the measured production yields listed in Table 4.

**TABLE 5:** Measured beam intercepts for the proton and deuteron beams.

	Incident particle	% beam intercept
Original puller	Proton	46 ± 1
Original puller	Deuteron	53 ± 5
New puller	Proton	55 ± 3
New puller	Deuteron	54 ± 5

**FIGURE 6:**
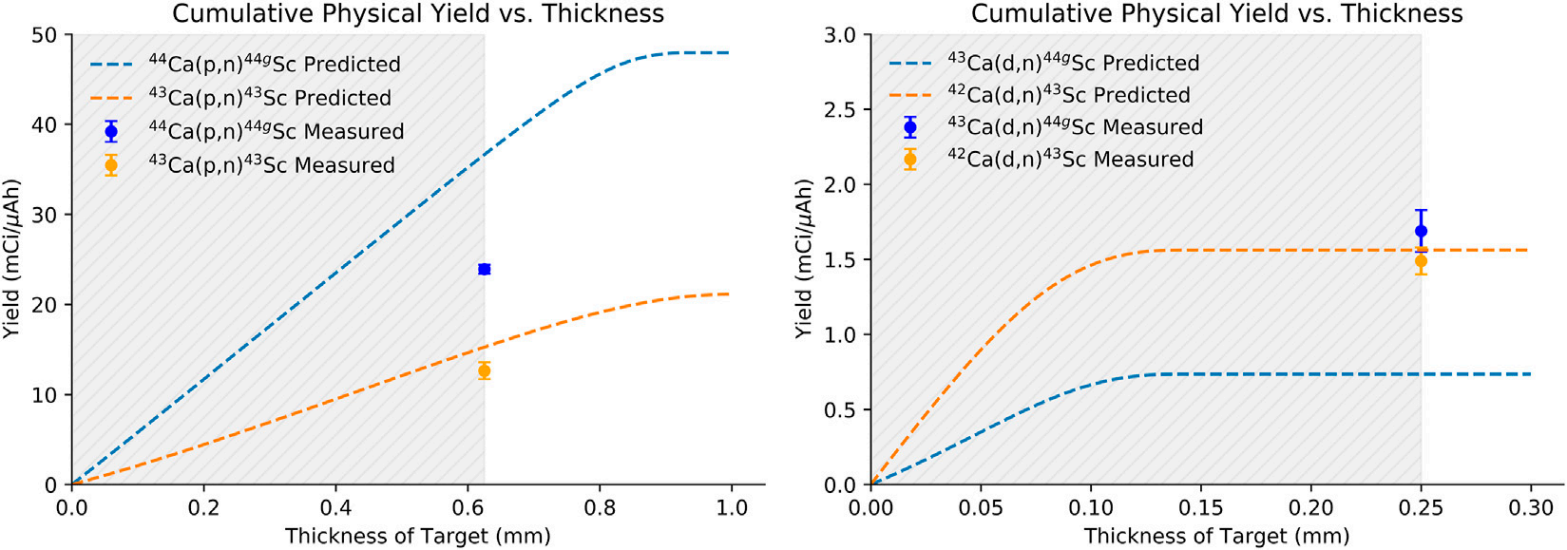
Measured production yields compared to literature reported cross sections fitted to TENDL_19 predicted curves for 13.6 MeV incident protons (left) and 5.8 MeV incident deuterons (right) (de Waal, et al., 1971b; Levkovski, 1991; Koning et al., 2012; Krajewski, et al., 2013; Carzaniga, et al., 2017; Carzaniga, et al., 2019).

Figure 6 shows the predicted physical yields versus target thickness for a 13.6 MeV proton (left) and 5.8 MeV deuteron beam (right). Dashed lines are predicted yields taken from fits to published cross section values. The fitted cross sections used for this calculation are shown in blue in Figure 2. Experimentally measured, beam-spot corrected yields are additionally shown in Figure 6. Experimentally measured yields are similar to, but slightly lower than, predicted yields except in the case of the ^43^Ca(d,n)^44g^Sc reaction where measured yields were higher than predicted. Predicted yields were based on cross-sections reported by Carzaniga (Carzaniga, et al., 2017; Carzaniga, et al., 2019) except for the ^43^Ca(d,n)^44g^Sc reaction where the only experimental data available was reported in (de Waal et al., 1971a). For the other deuteron-induced reaction reported in (de Waal et al., 1971b) [^42^Ca(d,n)^43^Sc], the cross sections were lower than those reported by Carzaniga et al. (2019) for the same energy range. The discrepancy between cross sections reported by Carzaniga and de Waal are likely the cause of the difference in trends between the experimentally measured and predicted yields observed in this work.

**FIGURE 7:**
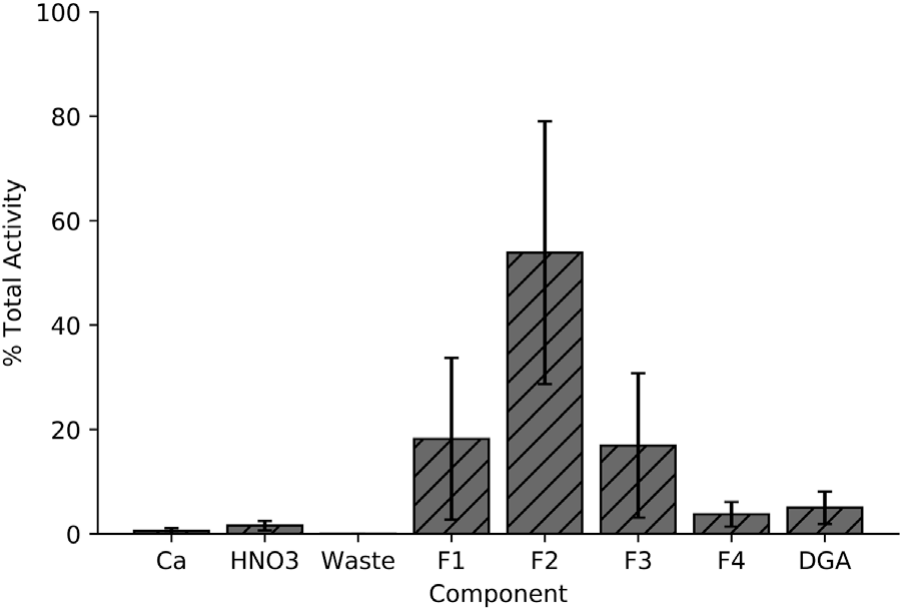
Elution profile for the Ca^+2^/Sc^+3^ radiochemical separation used in this work. Activity distribution in each of the 4 eluted fractions varies with each experiment, but the overall eluted fraction remains consistent.

Figure 3 illustrates the separation strategy for isolating the Sc^+3^ ions from the Ca^+2^ ions. The low affinity for Ca^+2^ ions on the branched DGA resin permitted efficient recycling from the 5 mL load and 10 mL 6 M HCl rinse stages of the separation (Alliot, et al., 2015). Other +2 metals (such as Cu^+2^) are also rinsed from the resin in this step. The 1 M HNO_3_ wash removes metals such as iron and zinc due to the low affinity for these ions in 1 M HNO_3_ and minimal loss of Sc^+3^ was observed as seen in the elution profile (Figure 7) (Alliot, et al., 2015). Elution with 2 mL of 0.01 M HCl effectively removes 92.8% ± 4.0% of the loaded scandium. However, there is large variation in the activity that is in each 0.01 M HCl elution fraction (*n* = 10), perhaps due to variations in volumes of liquid being used to elute the activity (residual liquid may be left in tubing or on walls in parts of the system)*.* Following a 1 h, 5 μA, 13.6 MeV proton irradiation of a 150 mg ^44^CaO target, this chemical isolation procedure isolates 1.4 ± 0.3 GBq (38 ± 9 mCi) of ^44g^Sc with 99.5% ± 0.05% radionuclidic purity in 2 mL 0.01 M HCl 2 h after EoB.

### 3.2 Recovery of enriched calcium

^42/43/44^Ca was recovered in the form of ^42/43/44^CaCO_3_ through slow addition of (NH_4_)_2_CO_3_ and converted back to ^42/43/44^CaO through decarboxylation in a vertical tube furnace (900°C for 60–90 min) for future irradiations. The enriched ^42/43/44^CaCO_3_ target material was recovered with an efficiency of 95% ± 2% (*n* = 20). If argon gas is passed over the graphite crucible during the decarboxylation procedure, the crucible surface remains smooth and losses from transferring and heating are <1%.

### 3.3 Apparent molar activity measurements and trace metals analysis

**FIGURE 8:**
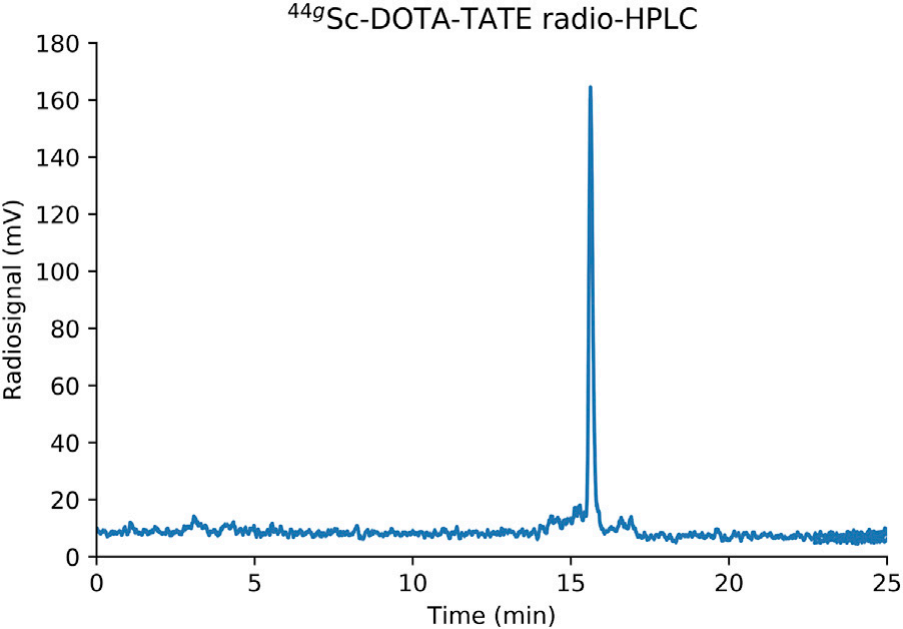
Radio-HPLC chromatogram of ^44g^Sc-DOTA-TATE.

The quality of the final ^43/44g^Sc eluent was determined through titrimetric reactions with the chelator DOTA and the ability to label with the DOTA-based peptide DOTA-TATE. Molar activity of ^43/44g^Sc produced through a 30–60 min, 13.6 MeV proton energy and 5 μA current irradiation of either ^43^CaO or ^44^CaO targets was 48 ± 11 GBq/μmol (1.3 ± 0.3 Ci/μmol) (*n* = 3) at end of chemistry. Additionally, 37 ± 5 MBq (1 ± 0.14 mCi) aliquots of ^43/44g^Sc were labeled with DOTA-TATE at a molar activity of 7.14 MBq/nmol (0.2 mCi/nmol) and assayed using HPLC. Radio-HPLC chromatograms (Figure 8) show near quantitative labeling (>98%). Free ^43/44g^Sc eluted with the solvent front (4 min) and ^43/44g^Sc-DOTA-TATE eluted at 15.6 min.

Amounts of Ca and commonly seen metals such as Fe, Zn, and Cu that may affect Sc-labelling were measured through MP-AES and are listed in Table 6 (*n* = 5). The efficient removal of metals such as Fe, Zn, and Cu in the radiochemical isolation strategy resulted in the high apparent molar activity observed with Sc and DOTA. The mass of Ca that was co-loaded on to the column and seen in the eluent resulted in minimal enriched material loss (<1 mg).

**TABLE 6:** Trace metals analysis of an eluted fraction of ^43/44g^Sc using MP-AES.

Element	Impurities (ppb or μg/L)
Iron	0.7 ± 0.9
Zinc	1.7 ± 1.3
Copper	1.4 ± 0.1
Calcium	226.5 ± 45.6

### 3.4 Clinical phantom scans

**FIGURE 9:**
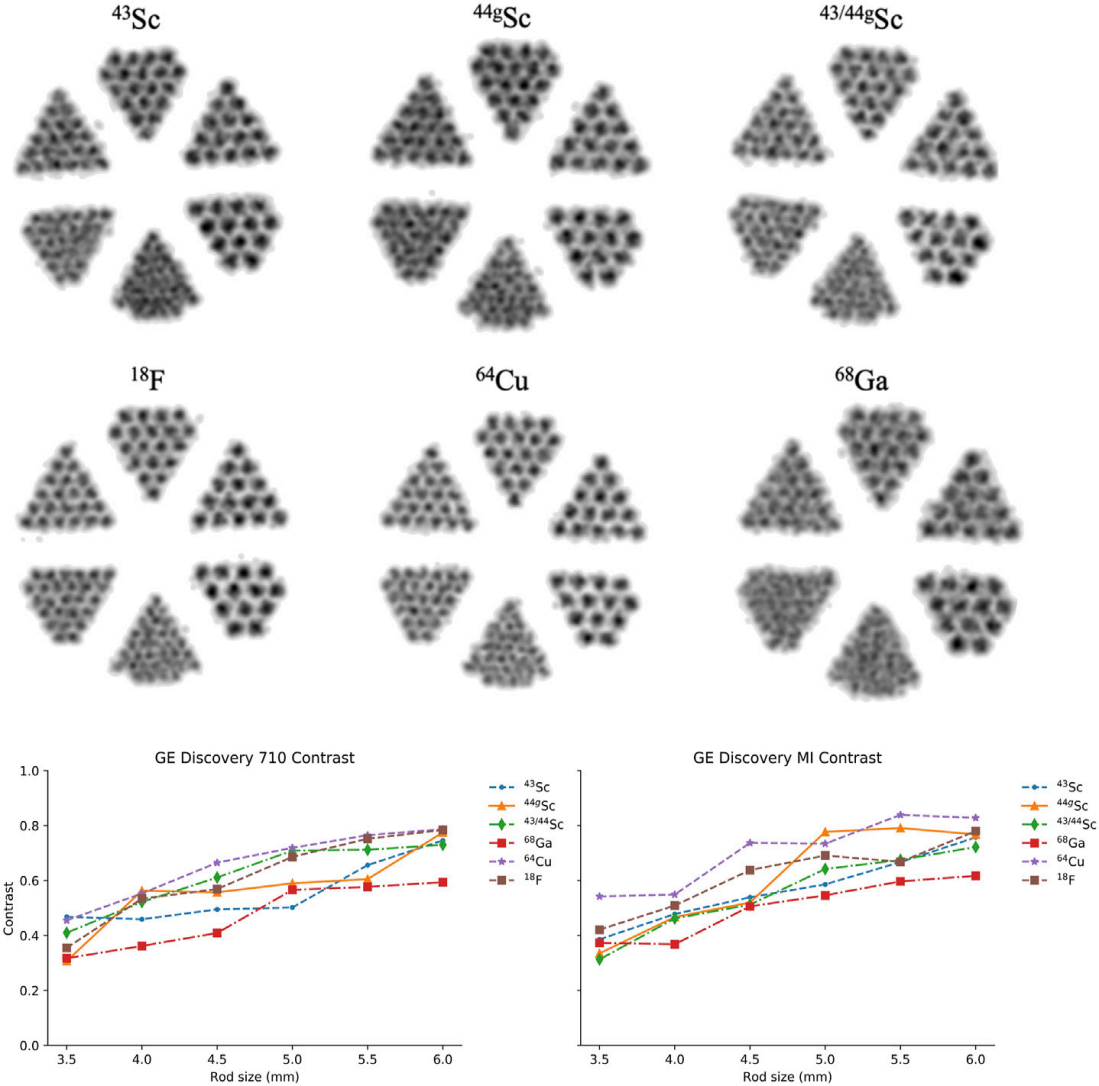
Reconstructed PET scans of the Derenzo phantom filled with each radionuclide on the GE Discovery 710 (top). Comparison of contrast ( $C_{rod}$ ) for each radionuclide investigated on both scanners (bottom).

The contrast, C_rod_, was compared between the 6 radionuclides on the two PET/CT scanners. Figure 9 shows a cross-sectional image of the reconstructed Derenzo phantom on the GE Discovery 710 (similar images were seen on the GE Discovery MI). Qualitative differences can be seen between shorter-range positron-emitters such as ^64^Cu and ^18^F compared to the higher-range positron-emitter ^68^Ga. All six sections of rods are easily distinguishable for ^64^Cu and ^18^F while the lower diameter rods (3.5 and 4 mm) are significantly blurred in the ^68^Ga image. Radioisotopes of scandium demonstrate image quality between the low and high positron range radionuclides where the smallest diameter rods are indistinguishable, but resolution improves with the 4 mm diameter rods. The calculated contrast values are shown in Figure 9 as well.

On both scanners, ^64^Cu and ^18^F demonstrated the greatest contrast across the varying rod diameters while ^68^Ga performed the worst across all rod diameters which is consistent with what we would expect from the positron energies listed in Table 2. Between ^43^Sc, ^44g^Sc, and ^43/44g^Sc, there is no significant difference in contrast, suggesting minimal benefit in contrast from ^43^Sc’s softer positron energy using these clinical PET/CT systems.

## 4 Discussion

The goal of this study was to compare production yields, radionuclidic purity, and PET image quality between ^43^Sc and ^44g^Sc. Among the six reactions that produce ^43^Sc and ^44g^Sc with protons and deuterons on small cyclotrons, the ^44^Ca(p,2n)^43^Sc and ^44^Ca(d,2n)^44g^Sc reactions are only possible above 15.2 MeV protons and 5.8 MeV deuterons, eliminating some commercial machines from consideration. The proton-induced reactions investigated here [^43^Ca(p,n)^43^Sc and ^44^Ca(p,n)^44g^Sc] had significantly higher production yields (by approximately a factor of 10) than the deuteron-induced reactions [^42^Ca(d,n)^43^Sc and ^43^Ca(d,n)^44g^Sc] but were performed with higher mass targets (approximately 150 mg versus 60 mg CaO) and higher incident energies (13.6 or 15.2 MeV protons versus 5.8 MeV deuterons). The peak of the cross-section curves for the deuteron-induced reactions were not fully captured with the energies and target thickness used here (Figure 2, gray area). Accelerators capable of accelerating higher energy deuterons would be able to produce higher yields through these routes. Thick target yields for the proton-induced reactions were not investigated in this work due to the large masses (∼250 mg CaO) required to degrade the proton energy in the target from 13.6 MeV to 4 MeV where the cross section approaches zero (Carzaniga, et al., 2017).

Due to the relatively low isotopic purity of available ^43^Ca (83.93%), an EoB radionuclidic purity of only 87.81% ± 0.03% ^43^Sc was achievable through proton bombardment of ^43^Ca. The main radioisotopic contaminants from this reaction are ^44g^Sc at 12.16% ± 0.03% total activity and ^48^Sc (t_1/2_ = 43.71 h; β^-^_mean_ = 220.5 keV; β^+^_intensity_ = 100%) at 0.02% ± 0.01% total activity at EoB. The production of ^44g^Sc results from a (p,n) reaction on the 5.06% ^44^Ca present in the enriched ^43^Ca target material and could be minimized through use of higher purity ^43^Ca target material. With 100% isotopically pure ^43^Ca, ^44g^Sc would be removed and ^43^Ca(p,n)^43^Sc measured yields would increase to 288.6 MBq/μAh (7.38 mCi/μAh), or 557.2 MBq/μAh (15.06 mCi/μAh) when corrected for beam intercept. Considering the alternative deuteron-induced reaction, ^42^Ca(d,n)^43^Sc, the radionuclidic purity at EoB was 99.36% ± 0.05% ^43^Sc at EoB with no co-production of ^44g^Sc’s metastable state ^44m^Sc. However, this route will require longer irradiation times and a cyclotron capable of accelerating deuterons to produce the same EoB activity as the proton-induced route.

For producing ^44g^Sc, the ^44^Ca(p,n)^44g^Sc route results both in higher production yields and greater radionuclidic purity than the ^43^Ca(d,n)^44g^Sc route with the conditions investigated in this work. This is partially due to the low purity of the ^43^Ca and the smaller mass of the target. Cyclotrons capable of accelerating higher energy deuterons (>8 MeV) along with higher purity and thicker ^43^Ca targets would increase the production yields compared to those reported here. However, the higher predicted cross sections for the ^44^Ca(p,n)^44g^Sc reaction compared to ^43^Ca(d,n)^44g^Sc (Figure 2) suggest that the highest yields of ^44g^Sc would still be through proton bombardment of ^44^Ca. The presence of longer-lived radioisotopes of scandium such as ^46^Sc are also comparable in both reaction routes in the target thickness and energy range investigated herein: At EoB, there was 0.007% ± 0.003% ^46^Sc for ^44^Ca(p,n)^44g^Sc and 0.013% ± 0.003% ^46^Sc for ^43^Ca(d,n)^44g^Sc.

When considering translation to clinical studies, long-lived radioisotopes of scandium (^46^Sc, ^47^Sc, and ^48^Sc) should be minimized to avoid patient dose and handling of radioactive waste (De Nardo, et al., 2021). The threshold for ^68^Ge (t_1/2_ = 270.8 days) impurity set by the European Pharmacopoeia in ^68^Ge/^68^Ga generator elutions is 0.001% (Council of Europe, 2013, 2013). The presence of ^46^Sc (t_1/2_ = 83.79 days), ^47^Sc (t_1/2_ = 3.3492 days), and ^48^Sc (t_1/2_ = 43.71 h) for the reaction routes at the irradiation parameters investigated herein are lower than the 0.001% ^68^Ge limit at EoB with the exception of ^43^Ca(p,n)^43^Sc which results in 0.0012% ^48^Sc at the end of bombardment.

Radiochemical isolation with branched DGA resin was procedurally simple and achieved efficient target recycling and high radiochemical yields [95% ± 2% enriched calcium recovery (*n* = 20) and 92% ± 4% ^43/44g^Sc activity in 2 mL 0.01 M HCl (*n* = 10)]. From the calcium mass that was measured via MP-AES, the Ca/Sc separation factor is on the order of ∼10^5^. Quantitative labeling with DOTA-TATE and high apparent molar activities with DOTA were additionally achieved with this separation strategy and minimal trace metals were observed through MP-AES analysis.

Because PET resolution is impacted by positron range (energy), ^43^Sc may have better PET resolution than ^44g^Sc. Additionally, it has been suggested that ^44g^Sc’s higher energy gamma (1,157 keV; 99.9%) as opposed to ^43^Sc’s lower energy gamma (373 keV; 22.5%) may contribute negatively to image quality due to the potential to scatter within a PET scanner’s 511 keV window (Carzaniga, et al., 2017) and negatively to patient dosimetry. In this study we observed no significant difference in the contrast of the phantoms filled with ^43^Sc, ^44g^Sc, or the mixture ^43/44g^Sc.

## 5 Conclusion

This work demonstrated that isotopically enriched CaO targets can be used to produce high yield and high radionuclidic purity ^43^Sc and ^44g^Sc through proton and deuteron bombardment. While several reaction routes produce either ^43^Sc or ^44g^Sc, laboratory capabilities, circumstances, and budgets are more likely to dictate which reaction route is chosen. When comparing PET image quality, ^43^Sc and ^44g^Sc contrast were comparable, and in some cases favorable compared to other conventional PET radionuclides. In addition, no significant difference in contrast was observed due to the lower energy of ^43^Sc’s positron compared to ^44g^Sc. These results indicate that, while ^44g^Sc’s higher energy gamma emission and co-produced metastable state (^44m^Sc) may have dosimetric consequences for in-human studies in the future, with regards to yields, radionuclidic purity, and image contrast, ^44g^Sc has superior or comparable characteristics compared to ^43^Sc.

## Data Availability

The raw data supporting the conclusion of this article will be made available by the authors, without undue reservation.

## Footnotes

1 Kretowicz, M. N. (2023). *Promising Scandium-44 radionuclides for nuclear medicine from a ^44^Ti/^44g^Sc generator*.
